# Development and Implementation of a Fully Customised System for Monitoring a Long-Span Cable-Stayed Bridge Undergoing Rehabilitation Works

**DOI:** 10.3390/s26092786

**Published:** 2026-04-29

**Authors:** Catarina Oliveira Relvas, Giancarlo Marulli, Carlos Moutinho, Elsa Caetano

**Affiliations:** CONSTRUCT—ViBest—Faculty of Engineering (FEUP), University of Porto, R. Dr. Roberto Frias S/N, 4200-465 Porto, Portugal; up201800981@fe.up.pt (G.M.); moutinho@fe.up.pt (C.M.); ecaetano@fe.up.pt (E.C.)

**Keywords:** Structural Health Monitoring (SHM), stay-cable bridges, customized monitoring systems, edge computing, digital databases and interfaces, traffic loads and temperature effects on structures

## Abstract

This work explores the key capabilities of emerging sensing technologies in the context of Structural Health Monitoring (SHM) of civil infrastructures, aiming to contribute to research on integrated and intelligent systems for more accessible and efficient monitoring solutions. As a case study, it focuses on the analysis of the static and dynamic behavior of the Edgar Cardoso stay-cable bridge during its rehabilitation, using fully customized transducers and equipment. The developed system integrates sensors capable of measuring accelerations, displacements, and temperature, which are connected to an autonomous data acquisition and transmission network. A digital interface was also developed to store, process, and visualize the collected data, enabling remote access for subsequent interpretation and analysis. The main contribution of this research lies in the use of optimized wireless monitoring systems with extended autonomy. This is achieved by employing edge computing techniques to minimize energy consumption during data transmission, as well as by managing the sleep modes of the sensor nodes. At same time, a methodology was proposed for the automatic and real-time estimation of axial forces in cables. This approach relies on the use of innovative edge computing tools, combined with the taut string theory as a simplified modelling framework. The results confirm the effectiveness of the developed system in achieving long-term operation without compromising monitoring performance. In addition, the developed system enabled the identification of the structure’s dynamic properties, particularly natural frequencies. The temperature profiles in critical sections, as well as displacements in the expansion joint were also measured and evaluated. The results demonstrate the potential of customized sensing solutions as effective tools for the management, maintenance, and long-term preservation of strategic infrastructures.

## 1. Introduction

Structural monitoring in Civil Engineering plays a key role in the safety, durability and efficient management of infrastructures, enabling the early detection of anomalies and the implementation of preventive maintenance strategies. Traditionally, bridge inspection has relied on manual methods, which, while effective, are often time-consuming, labour-intense, and subjective to human error [1,2]. As a result, there has been a growing interest in the development and implementation of Bridge Health Monitoring (BHM) systems, which utilize advanced technologies to continuously assess the condition of bridge structures in real time in ensuring the safety and longevity of bridge infrastructures worldwide [3,4,5].

The emergence of new generation detection technologies (NGs) represents the cutting edge of sensor innovation, developed to transcend the limitations inherent in conventional detection systems [6,7]. Furthermore, the advancement of sensor technologies must be accompanied by the evolution of the monitoring systems themselves, promoting the development of more integrated and intelligent solutions that align with the rapid progress of computational and information technologies [8,9].

New generations of wireless sensors are revolutionizing structural monitoring by offering improved energy efficiency, enhanced connectivity, and reduced costs. The implementation of sensors without damaging the structure is achieved through non-intrusive techniques, such as adhesive-mounted wireless sensors and remote data transmission, ensuring seamless integration with existing infrastructure. This constitutes one of the main advantages of wireless sensors, as they avoid the logistics of spreading cables on structures, especially in large structures or in existing structures where it is even more difficult to accommodate long cables.

However, despite the advantages offered by wireless systems, there remains significant resistance to their adoption in structural health monitoring applications. As a result, many actual monitoring projects are still recurring to wired conventional systems, as wireless systems still present certain limitations, particularly regarding power supply in standalone configurations, which can impact both the cost and reliability of the overall system.

The problem of powering the wireless sensors is that, although they provide an attractive solution by eliminating extensive cabling and facilitating the installation process, they still require a reliable power source. If electrical cabling is needed to supply power to each sensor, the advantages of wireless communication become significantly diminished, as traditional wired systems would then represent a more straightforward solution. Consequently, the deployment of truly wireless systems requires autonomous power sources, such as batteries of local solar panels. However, this requirement introduces additional costs, which may compromise the benefits of wireless technology. The problem becomes more critical in applications requiring continuous data transmission, such as accelerometer-based monitoring for Operational Modal Analysis with high sampling rates. In such cases, the associated energy consumption increases substantially, necessitating larger and more expensive power supply systems.

It is within this context that the work presented in this paper is framed, as it follows an approach aligned with recent trends in wireless monitoring systems. Specifically, instead of continuously transmitting raw data to a remote server for processing, the proposed approach performs data processing locally at the sensor level, known as Edge Computing [10]. In this case, only a set of locally computed structural parameters is transmitted, rather than complete datasets, resulting in a significant reduction in the volume of information. Consequently, communication can be performed intermittently, for example, at hourly intervals, requiring only short transmission periods. Under these conditions, battery-based power supply systems can sustain sensor operation for very long periods. In addition, the customized sensors used is this work, leverage low-power communication protocols, to enable long-range data transmission with minimal energy consumption [11,12,13]. Advanced materials and miniaturization techniques have increased sensor durability and sensitivity, making them more effective in harsh environments [14].

The developed monitoring system integrated new sensing techniques such as Internet of Things, Digital Twin Framework and Multisensory Data-fusion. The designed system not only optimizes sensor solutions to meet the specific requirements of the practical case but also incorporates a strong digitalization component. This digital dimension is achieved through the development of an integrated real-time monitoring platform supported by a database hosted on a web server, ensuring secure and centralized data management [15,16,17]. The system allows continuous acquisition, storage, and processing of information collected by the sensors, promoting efficient data organization and long-term accessibility. In addition, a dedicated graphical interface has been developed to facilitate data visualization and interpretation, allowing for clear and intuitive remote consultation of structural parameters. This integration between hardware and digital infrastructure represents an important step towards the implementation of intelligent monitoring systems capable of supporting decision-making processes in maintenance, safety assessment and structural management.

In this context, the main objective of this paper is to contribute to the development of optimized wireless monitoring systems with extended autonomy by employing edge computing techniques to minimize energy consumption during data transmission, as well as by effectively managing the sleep modes of sensor nodes. This was achieved by demonstrating how the proposed architecture can be used to estimate the natural frequency of a stay cable using a wireless sensor integrating advanced Edge Computing techniques, without the need to transmit the complete time signal to a remote receiver. On the other hand, Edge Computing was also implemented in other sensors to locally calculate several parameters of the structural response. Among these, the determination of RMS values proved to be a demanding task, given the limited computational capacity of low-power microcontrollers to deal with large numbers.

On this basis, this document describes the development and implementation of a fully customized monitoring system based on the principles outlined before, highlighting their potential to provide efficient and reliable performance while reducing the complexity and cost of conventional monitoring solutions. In addition, several results extracted from the signals collected in the monitoring campaign are presented, including the identification of the main natural frequencies of the suspension cables and the deck through spectral analysis, the detection of traffic-related patterns from acceleration data, and the estimation of cable stresses using the theory of stretched strings over a period of one month [18]. The influence of environmental factors, particularly temperature variations and expansion joint displacements, are also depicted. Similar dynamic monitoring approaches have been successfully applied in large bridge structures, such as the Dom Luís I Bridge in Porto [19], further validating the effectiveness of customized sensors and the developed platform as valuable tools for the management and maintenance of complex infrastructure.

## 2. Description of the Bridge

**Figure 1:**
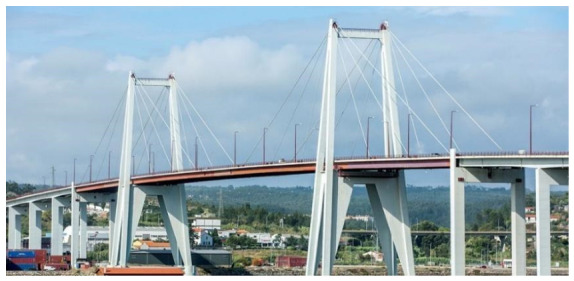
General view of the Edgar Cardoso Bridge in Figueira da Foz.

**Figure 2:**
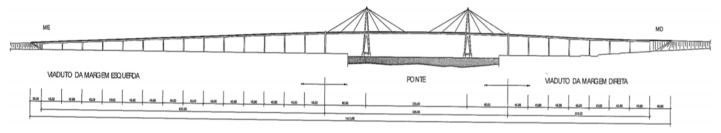
Side view of the Figueira da Foz Bridge, designed by Professor Edgar Cardoso and completed in 1982 [20].

The case study adopted in this research is the Edgar Cardoso Bridge, a landmark structure in Portuguese engineering, representing the country’s first major cable-stayed bridge, inaugurated in 1982 (Figure 1 and Figure 2). Situated in the city of Figueira da Foz, Coimbra District, the bridge crosses the Mondego River, linking the northern parish of Buarcos e São Julião to the southern parish of São Pedro. Commonly known as Ponte da Figueira da Foz, it was designed by the distinguished Portuguese Engineer Edgar Cardoso.

**Figure 3:**
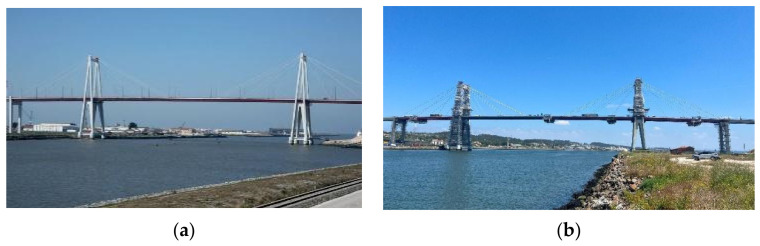
Photograph of the Edgar Cardoso Bridge: (**a**) before rehabilitation; (**b**) under rehabilitation.

The Edgar Cardoso Bridge is currently undergoing rehabilitation, marking its second major intervention since inauguration (Figure 3). The first significant maintenance work took place in 2005, when structural reinforcements were carried out. Nearly 20 years later, the bridge requires a new intervention to ensure safety and longevity, primarily due to the progressive degradation of the cable suspension system, evidenced by increasing wire breakage. In addition, the deck’s metal elements, particularly at the connections with the concrete slab, show significant deterioration. Consequently, rehabilitation of this area has also been included in the repair plan.

**Figure 4:**
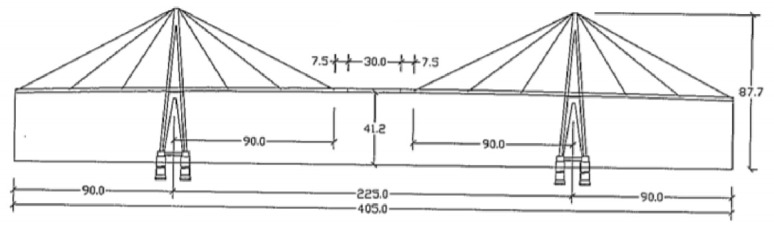
View of the cable-stayed bridge [20].

The Edgar Cardoso Bridge has a length of 405 m, 225 m in the central section and 90 m in the lateral span (Figure 4). The deck, made of steel and concrete, is supported by two self-supporting towers, two transition pillars and six pairs of cables. The free height above the water level is around 40 m with a maximum column height of approximately 80 m. The retaining ties are anchored to the deck, which in turn is anchored to the transition columns. The deck also has an isostatic central Section 30 m long supported on the south side by fixed supports and on the other side by movable supports.

The bridge deck is made up of a combination of steel and concrete. As far as longitudinal development is concerned, the deck has two joints in its middle area, which delimit a simply supported central span 30 m long.

**Figure 5:**
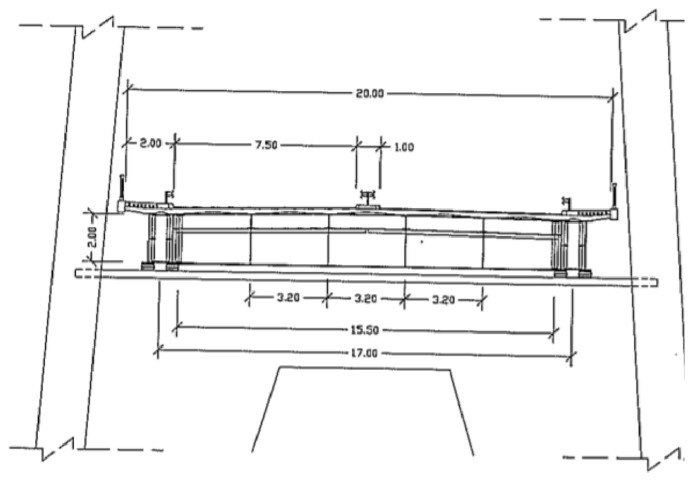
Cross-section of the deck [20].

The transverse direction is 20 m wide (Figure 5) divided into four traffic lanes, a central divider and two sidewalks, one on each side. The platform is made up of a mixed steel–concrete structure in which the metal elements form an orthotropic system with modules 10 m long and 17 m wide. The wired concrete slab rests on the stiffening beams and stringers and consists of two cantilevers at the ends, two small spans of 0.50 m between the twin elements of the stiffening beams and five spans of 3.20 m between the stringers. Its thickness varies according to the parabolic law that defines the progress of the lower face. The thickness of the slab is 0.20 m on the supports and 0.13 m in the spans.

**Figure 6:**
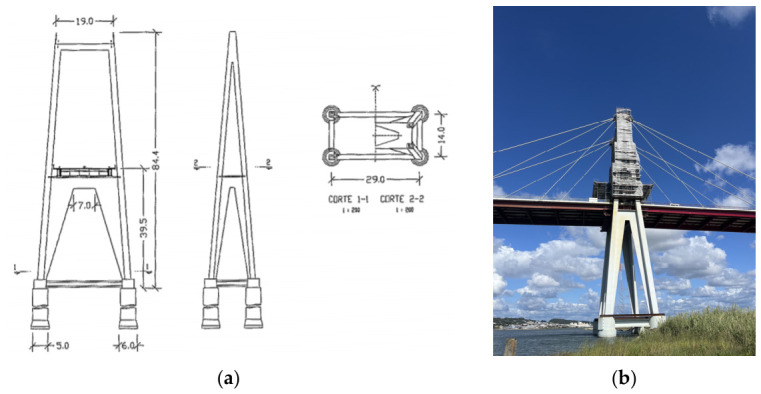
(**a**) Geometry and dimensions of the Figueira da Foz Bridge towers [20]; (**b**) Photograph of the north bridge tower under rehabilitation.

The bridge has two concrete pylons with 84.4 m to support the vertical reactions (Figure 6). Each pylon has four concrete legs with hollow squared section, inclined longitudinally and transversely, connected at the top by a transversal beam and under the deck by a concrete laminate plate. At the base level, the towers are supported by four circular hollow piles, with an exterior diameter of 5 m and wall thickness of 0.4 m. The pile blocks are interconnected by prestressed I-sections concrete beams.

**Figure 7:**
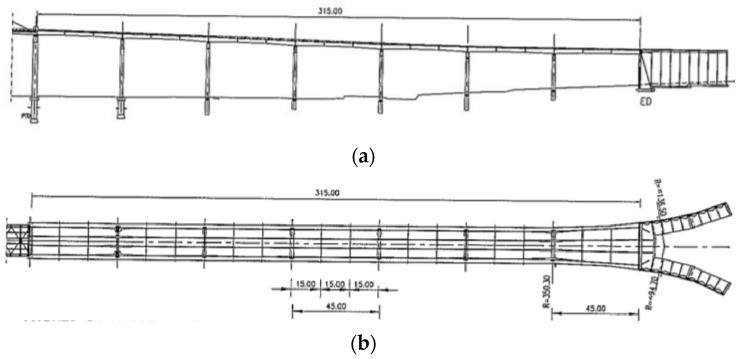
General representation of the right viaduct of the Edgar Cardoso Bridge [20]: (**a**) Right bank viaduct longitudinal section; (**b**) Right bank viaduct plan view.

The viaducts are made up of beamed slab decks, each containing 4 longitudinal beams separated by 5.2 m, with continuous spans of 45 m supported on hollow pillars, whose heights vary between 13 and 34 m (Figure 7).

The beams, which vary in height from 2.3 m to 2.8 m, are prestressed longitudinally. In contrast, the slab, which varies in thickness from 0.22 m to 0.18 m, is prestressed transversely. The beams are braced transversally every 15 m.

The viaduct pillars consist of two hollow rectangular columns, joined at the top by a hollow beam. The beams are supported on the pillar crosspieces by means of bolts and lead braces. At the transition pillars, the viaducts are supported by movable roller-type metal devices with guides. At the abutments, the viaducts are supported by strapped neoprene braces equipped with a Teflon sliding plate, installed during the rehabilitation work. In addition, to control the effects of seismic action, viscous dissipators were installed between the decks and the abutments. Although all the drawings represent the viaduct on the right bank, the same structural configuration and considerations apply to the viaduct on the left bank.

**Figure 8:**
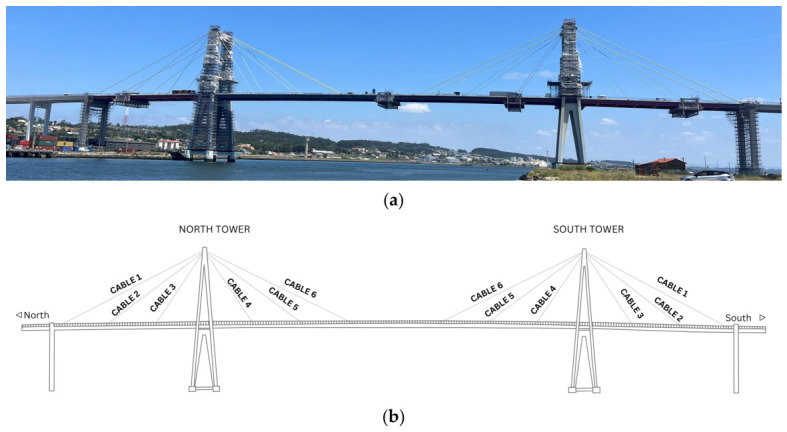
(**a**) Photograph of Edgar Cardoso Bridge; (**b**) General representation of the Edgar Cardoso Bridge.

The suspension cables are arranged in a fan shape, symmetrically on each side of the towers and are tied to the deck’s stiffening beams, 30 m apart (Figure 8). The cables are continuous between the central span and the lateral span, and the towers are equipped with saddles at the top.

The stay cables are made up of parallel steel wires: 109 strands are used on cables 1/6, 61 strands in intermediate cables 2/5, and 37 strands on cables 3/4. Each cable is protected by a galvanized and painted steel wire winding.

## 3. Development of Customised Sensors

**Figure 9:**
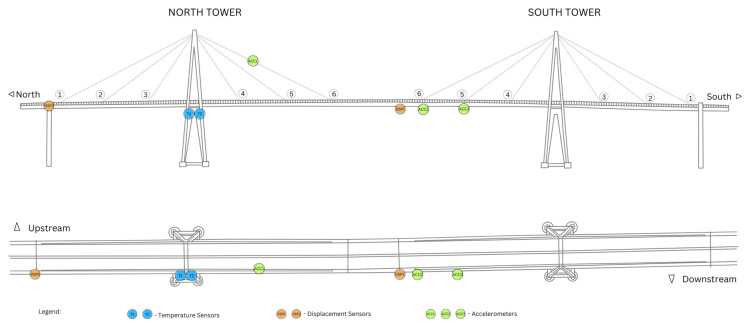
Instrumentation plan location.

In this monitoring program, data was collected from seven transducers (Figure 9). As part of the installation, the structure was equipped with three accelerometers, two temperature sensors, two displacement sensors and one reception station ensuring comprehensive data acquisition for analysis.

For consistency and clarity throughout this document, the accelerometers will be referred to as **ACC**, the temperature sensors as **T**, the displacement sensors as **DSP**, and the receptor sensor as **R**, as presented in Table 1.

One of the objectives of this project was to monitor the structure using autonomous wireless sensors capable of transmitting data without cabling, thereby efficient communication without physical constraints. In short, the installed sensors transmitted information remotely to the reception station located at the company facilities near the construction site, where the data were recorded locally and simultaneously sent to the database. The full features of the system will be described in detail in the following sections.

The sensors developed for this monitoring system are autonomous, requiring no wiring and enabling flexible installation. Compact in size, they were designed and built in the university’s laboratories, ensuring high precision and reliability. Each sensor was fully customized to the specific characteristics of the object under study, providing optimized integration and an appropriate response to the monitoring requirements.

### 3.1. Accelerometers

**Figure 10:**
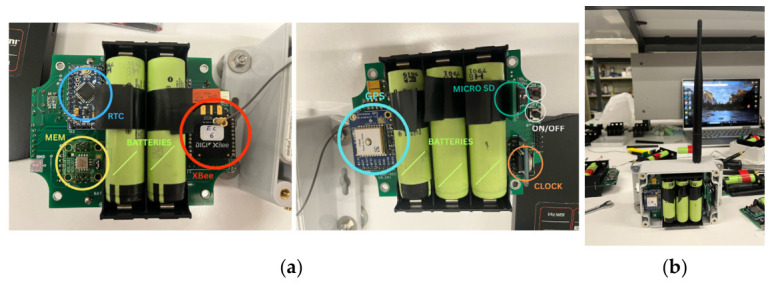
Accelerometer module: (**a**) internal components and (**b**) casing module.

The accelerometers are composed of an Arduino-based microprocessor that controls the entire process of data acquisition (Figure 10). The accelerometer used is a micro-electro-mechanical system (MEMS) type, namely the ADXL355 from Analog Devices Company. It has programmable sampling rates and programmable bandpass digital filters. The model used for the Edgar Cardoso Bridge incorporates an XBee radio to transmit data to the receiver, with the collected data also being stored locally on a microSD card to ensure robustness against potential data loss. This device accommodates five lithium batteries of 18650-type. The nominal voltage of each battery is 3.8 V, and the capacity is 3400 mAmps, ensuring an autonomy of approximately 2 months in continuous operation. The synchronization of the acquired signals between the different accelerometers is guaranteed by GPS.

The developed accelerometers are housed in a BUD PN-1323 external box made of polycarbonate, light grey in color, ensuring durability and protection. The enclosure has external dimensions of 90 mm (width) × 115 mm (depth) × 55 mm (height), providing a compact and secure structure for the components. The total weight of the unit is 0.6 kg, making it lightweight and easy to install.

The accelerometer ACC1 was installed on the cable with the aim of evaluating the installed axial force to which it is subjected and the vibrations levels of the cable 6. The device was placed on the cable-stay system attached on the top of the protective plastic sleeve, to not damage the cable, using metal chains. In this particular sensor, the microprocessor was changed to a Teensy 3.2 with higher power consumption than Arduino, leading to reduction in the expected autonomy to about 1 month.

On the other hand, accelerometers ACC2 and ACC3 were placed on the deck with the objective of identifying the dynamic parameters of the bridge deck. The accelerometer ACC2 was placed on the deck near the anchorage of cable 6 and the ACC3 device was placed near the anchorage of cable 5. The modules were fixed by magnetic attachment to the metal beam, without causing any damage to the structure.

Installing devices on the cable and on the deck made it possible to separate the natural frequencies of the bridge deck and the cable.

### 3.2. Temperature Sensors

**Figure 11:**
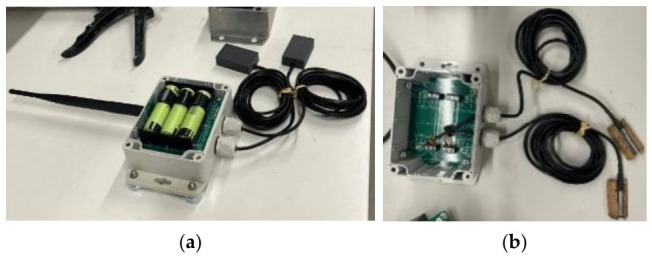
Temperature sensor: (**a**) view inside the module; (**b**) view of the BUD PN-1323.

The temperature sensors are the DS18B20 model from Maximum Integrated Company (Figure 11). The digital sensors measure temperatures from −55 °C to +125 °C, with a resolution of 0.125 °C and an accuracy of 0.5 °C from −10 °C to +85 °C, which is adequate for general applications in structural monitoring. No extra hardware is required, making this sensor simple and effective. The sensor has three-wire communication with the microcontroller. In addition, due to the unique 64-bit address, a high number of sensors can be used simultaneously through a single digital port on a microcontroller. In this application, the sensor was constructed using PTFE and stainless steel, materials that provide excellent durability and resistance to harsh environments. The sensor is equipped with two cables, ensuring stable connectivity and efficient data transmission. The temperature readings are acquired at 10-min intervals, with the data being stored locally on a microSD card and transmitted wirelessly to the receiver, after which the system enters sleep mode.

These modules are housed in a BUD PN-1323 enclosure with the same characteristics as the one used for the accelerometers, as described in Section 3.1. They are powered by three 18650 batteries, providing an autonomy of more than one year, which exceeds that of the accelerometers since the device operates in sleep mode for most of the time. The total weight of the unit is also 0.6 kg, ensuring it is compacted and simple to set up.

During the installation of the transducers, thermal paste was applied to ensure direct contact between the sensors and the measured element. This paste enhances thermal conduction from the steel to the sensors. The remaining area was insulated with cork to isolate the sensors from the ambient temperature.

The two sensors, and their housing, were fixed to the underside of the bridge deck without any exposure to sunlight. The first sensor, T1, was placed on the surface of the concrete structure and the T2 sensor was installed on the surface of the longitudinal steel beam. No drilling was required, just the use of glue. The installation of these devices was made possible using scaffolding in the north tower.

### 3.3. Displacement Sensors

**Figure 12:**
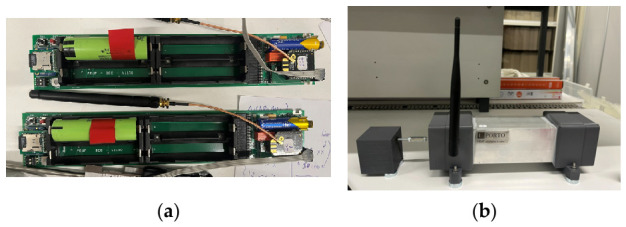
Displacement sensor: (**a**) interior board with batteries; (**b**) sensor casing.

The displacement sensor used is of the capacitive type and works inside an aluminum quadrangular cross-section with dimensions of 8 × 8 cm and length of 26 cm (Figure 12). The objective of using these devices is to evaluate the temperature effects on the structure, as well as to measure dynamic displacements originated by traffic loads.

The sensor is powered by four 18650-type batteries. Data access is provided through local storage on a memory card, with the Xbee radios (Digi International, Hopkins, MN, USA) transmitting the condensed information to a receiver each 10 min. In terms of autonomy, this device when operating in static mode (one reading each is 10 min), batteries may last more than 1 year. In dynamic mode (which was selected in this application), batteries ensure an autonomy of about 2 months. The dynamic mode was programmed to work with a sampling frequency 5 Hz. One of the significant features of this sensor is related to its ability to take measurements with 0.01 mm and precision in a range from 0 to 2 m. In practice, to prevent the sensor from being too long, the measuring range was adjusted to measure in the range of 0–150 mm.

Two displacement sensors were implemented on the structure. DSP1 was installed on the fixed support on the central section of the bridge, while the other sensor, DSP2 was placed on the north expansion joint. Both sensors were implemented on the downstream side.

### 3.4. Reception Station

**Figure 13:**
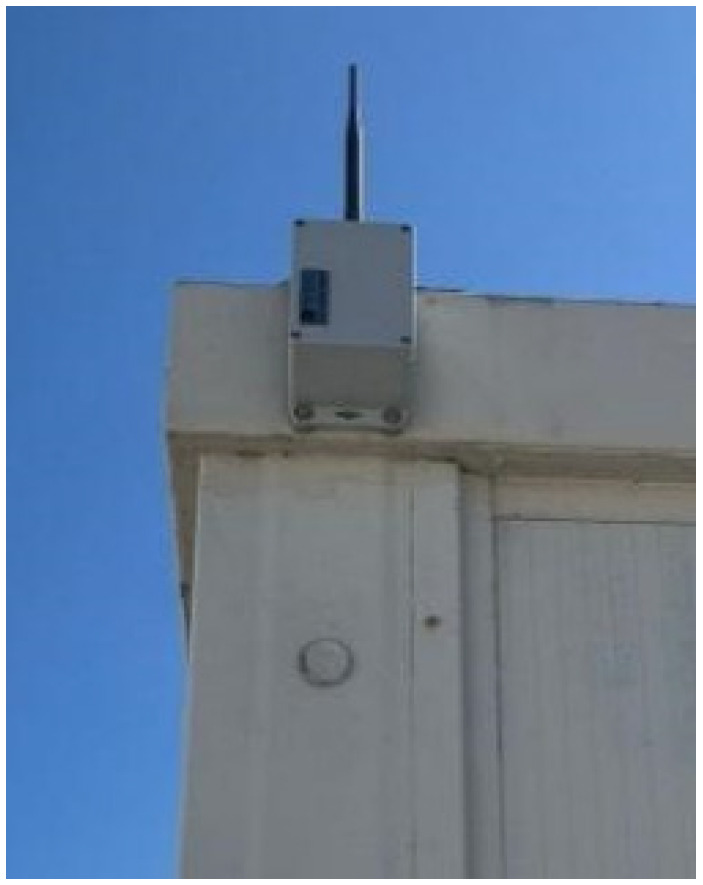
Receptor case at the company facilities.

The receptor, a battery-powered data reception station installed inside a BUD PN-1328 box, is dedicated to collect data sent by all sensors (Figure 13). This device has a Xbee radio to communicate with the sensors and has an ESP8266 module that provides an internet connection to send data to a database. The wireless network is provided by a local hotspot. The receiver was installed on the company facilities, receiving data from the various sensors with a time stamp of 10 min. This information is then saved locally and sent to an SQL database.

## 4. Monitoring System Architecture

**Figure 14:**
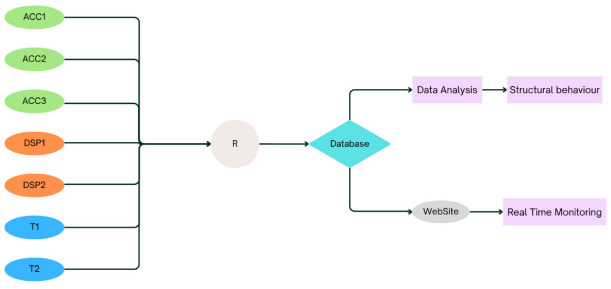
Schematic representation of the Structural Health Monitoring System.

As just described, the system is composed of multiple sensors, each one collects specific types of data and sends them to the reception station (Figure 14). This unit identifies and organizes incoming data from the different sensors, converts the sensor readings into a standardized format and sends the processed data to the database. The database acts as the core repository of the system. It stores all incoming data and enables further processing through two parallel pathways for analytical processing and for display on the website. The data is analyzed using algorithms and statistic tools. The database feeds information to the website interface, allowing engineers to monitor the structure in real time. The website provides visual dashboards and historical data.

### 4.1. Transmission of the Data

In order to reduce the amount of transmitted data, which can reach high volumes especially in the case of dynamic measurements, each local sensor was programmed using edge computing tools. Edge computing refers to a computational paradigm in which data processing is carried out locally at the sensor level, rather than relying entirely on remote servers or cloud infrastructures. This approach significantly reduces communication latency, minimizes bandwidth requirements, and increases the overall autonomy of the system.

As data is received from the seven sensors, a file structure is created in the receiver module to store the 46 distinct parameters collected from these sensors. Since data transmission between the sensors and the receiver is performed via XBee modules, it must be taken into account that this technology does not support the simultaneous reception of data from multiple sensors. Therefore, it is necessary to schedule the transmission time for each sensor, which is achieved by introducing a time delay for each transmission.

The complete process begins with the creation of a file at each 10-min interval in the receiver module. DSP1 then initiates its transmission 15 s after file creation, sending information such as battery voltage, along with the average, RMS, maximum, and minimum values of the measured signals. Similarly, DSP2 starts its transmission 30 s after the beginning of the 10-min interval, reporting parameters analogous to those transmitted by DSP1. This procedure is repeated for each sensor by introducing successive communication delays, ensuring that the various parameters collected by the sensors are transmitted sequentially. The complete sequence of time delays and transmitted parameters is presented in Table A1 (Appendix A).

### 4.2. Data Management

With regard to data management following acquisition by the receiver unit located at the construction site, the system architecture was designed to encompass the entire processing chain, from data storage to final user visualization.

The process begins with the deployment of a MySQL database hosted on a server. The database was specifically designed to accommodate the wide range of parameters collected during the monitoring campaign. The resulting scheme consists of 46 columns, each corresponding to a specific measurement parameter. The database structure was implemented directly in MySQL and managed using DBeaver (version 25.1x), which provides a graphical interface for organizing, visualizing, and handling tables and attributes.

Data collected by the receiver unit is transmitted to the server-hosted database, with PHP (versions 8.4) used as the backend language to manage the communication between the receiver and the database system. In addition, a Python (version 3.13) environment was configured to establish the database connection and automate the insertion of incoming data streams, ensuring reliable and continuous data storage.

Once stored, the data can be accessed and processed using MATLAB (version R2025a) for analysis and modelling, and Python for data handling and visualization tasks. An HTML-based website serves as the user interface, connecting directly to the database to retrieve and visually present the monitoring data to the end user.

After configuring the database infrastructure, the final step consisted of setting up the ESP-N platform and implementing the complete data transmission workflow, from the sensors to the receiver and subsequently to the database system.

**Figure 15:**
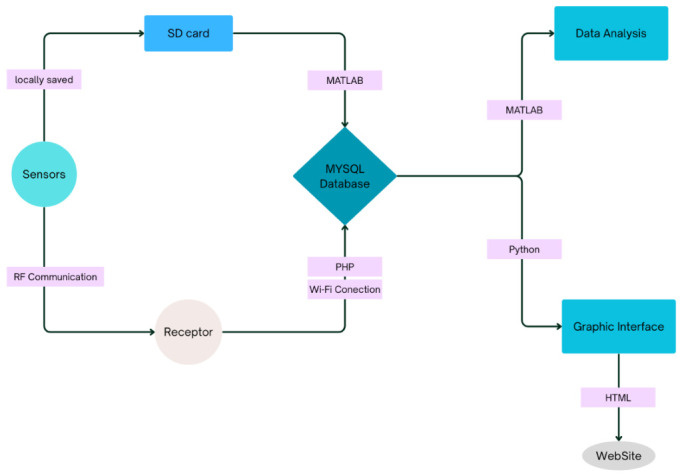
Flowchart of the monitoring system with sensors, showing the data flow from collection to the graphical interface and website.

The complete data management flow is illustrated in Figure 15.

### 4.3. Graphical Interface

**Figure 16:**
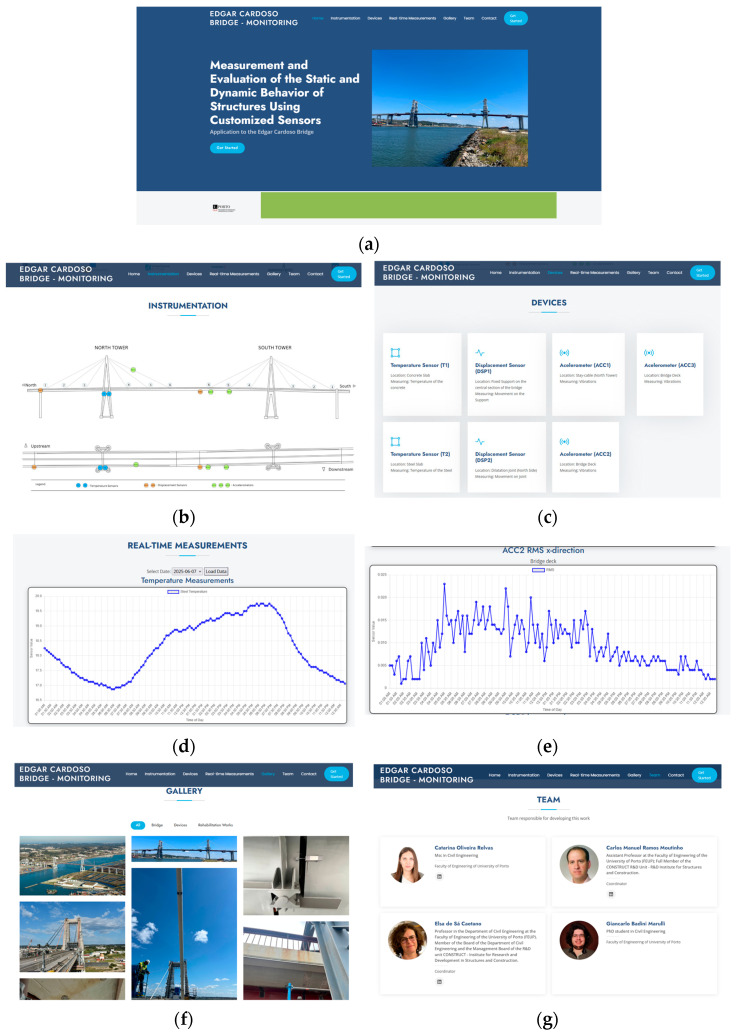
Web interface of the Edgar Cardoso Bridge Monitoring System: (**a**) Homepage; (**b**) Instrumentation layout; (**c**) List of services; (**d**,**e**) Real-time measurements; (**f**) Gallery of images from the monitoring system; (**g**) Team involved in the process.

The main objective in the development of the graphical user interface was to design a platform that is both visually appealing and modern, while maintaining a high level of accessibility and usability (see Figure 16). The intention was not only to provide an attractive and intuitive environment for the user, but also to ensure that the consultation of data and results could be performed in a straightforward and efficient manner, without the need for advanced technical knowledge. Particular attention was given to the balance between aesthetics and functionality, so that the interface could support a clear visualization of complex information while minimizing potential barriers to navigation.

In detail, the developed platform incorporates a dedicated section for structural instrumentation where the spatial distribution of the installed sensors is represented through a clear and interactive schematic of the bridge [21]. The main objective of this module is to provide users with an intuitive visualization of the monitoring system, allowing for a rapid understanding of both the type of sensors deployed and their precise locations.

Other module provides a comprehensive overview of the monitoring devices installed on the Edgar Cardoso Bridge, presenting each sensor individually with details regarding its location and the physical quantity measured. By structuring the information in this manner, the platform enhances clarity and ensures that users can quickly identify the function and role of each device within the overall monitoring system.

The Real-Time Measurements section of the platform is designed to provide continuous visualization of the data acquired from the installed sensors. In this module, measurements from devices such as battery voltage, temperature sensors, displacement sensors, and accelerometers are displayed in the form of interactive graphs that update dynamically, allowing the user to monitor structural responses as they occur. In addition to graphical representations, the system can also present the acquired data in tabular format, ensuring both a detailed numerical view and a more intuitive visual interpretation. This dual approach reinforces the analytical capabilities of the platform, enabling users to cross-reference real-time fluctuations with historical patterns and to extract meaningful insights for structural health monitoring.

The platform also includes complementary sections that enhance both contextual understanding and communication. The Gallery section provides a visual record of the instrumented areas and the overall structure under monitoring, offering users a direct perspective on the physical installation of the sensors. The Team section presents the members involved in the development and implementation of the project. Finally, the Contact section facilitates communication between users and the project team, ensuring accessibility for inquiries, collaborations, and future developments and information.

## 5. Customization of the Sensors for Advanced Monitoring

Customization allows the definition of specific software and hardware configurations, not only for the sensors but also for the operation of the entire monitoring system. This section outlines the key aspects that make the developed customized solutions attractive compared to commercial wireless systems, as well as the innovative tools adopted in sensor development.

### 5.1. Enhancing Autonomy

The main limitation hindering the widespread adoption of wireless monitoring solutions is the need to supply electrical power to sensors in order to ensure continuous operation. In commercial systems, high autonomy is typically achieved through the use of solar panels. However, these are often physically large and may not be suitable for all applications. Alternatively, battery-based solutions are commonly restricted to short-term monitoring, mainly in the case of dynamic measurements.

Nevertheless, if system energy consumption is properly optimized, batteries can become a viable long-term solution, provided that maintenance operations are appropriately planned. For instance, an autonomy period of six months to one year may be considered adequate, after which a scheduled site visit would be required for battery replacement and inspection of the equipment condition.

As an initial design decision, the selection of the sensor’s microcontroller must be carefully considered. If the objective is to minimize overall power consumption, low-power microcontrollers should be preferred. However, this choice inherently limits processing speed and computational capability. Platforms such as Arduino provide suitable solutions in this context. Conversely, if higher computational performance is required, for instance, through the use of platforms such as Raspberry Pi, power consumption becomes a critical issue. In the developed customized system, two types of microcontrollers were adopted, namely, a low-power Arduino-based platform for general sensors, such as temperature, displacement, and deck accelerometers, and a Teensy 3.2 microcontroller for the accelerometer module installed on the stay cables, where higher computational capability is required, as will be discussed later.

Another important design strategy is to take advantage of sleep modes for components that do not need to operate continuously. In the developed system, particular emphasis is placed on the use of the GPS module. Based on previous studies [22], it was concluded that in common Civil Engineering structure, where natural frequencies are relatively low, synchronizing the internal clock (DS3231 type) every six hours is sufficient, allowing the GPS module to remain in sleep mode practically all time.

Data transmission is also a critical aspect of the system. As previously mentioned, XBee radios were used to transmit data to a receiver unit located on-site. This solution leverages sleep modes, enabling the communication module to activate only during transmission periods, which typically last less than one second. To ensure proper operation, it is also necessary to coordinate the receiver’s wake-up schedule with the transmission intervals, as detailed in Appendix A of the document.

By adopting strategies such as those just described, the autonomy of the sensors can be significantly extended. In the case of sensors measuring static or quasi-static variables, such as temperature, autonomy can exceed one year. In fact, in previous long-term monitoring studies spanning several years, the batteries of temperature sensors were never depleted, and their actual autonomy was therefore never fully quantified. A similar situation is observed for displacement sensors operating in static measurement mode.

**Figure 17:**
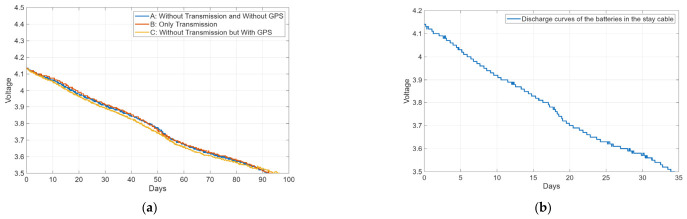
Discharge curves of the batteries: (**a**) Accelerometer module used in the deck; (**b**) Accelerometer module used in the stay cable.

In contrast, the scenario differs for accelerometers and displacement sensors operating in dynamic mode. Figure 17a presents, for example, the battery discharge curves of an accelerometer similar to that used on the bridge deck, equipped with five 18650-type lithium batteries with a total capacity of 17 Ah. Three curves are shown, corresponding to different operating conditions, namely with and without GPS usage and data transmission. As can be observed, when combined with effective sleep mode strategies, the use of peripherals such as GPS and data transmission has a negligible impact on overall energy consumption, which represents a remarkable achievement on enhancing autonomy. Additionally, it is observed that, in this configuration, the average battery life is approximately 93 days, meaning that this module consumes 7.6 mAh.

On the other hand, Figure 17b shows the discharge curve of an accelerometer module similar to that used in the stay cable. In this case, the use of six 18650-type lithium batteries, with a total capacity of 20.4 Ah, provided an autonomy of approximately 34 days, corresponding to an average consumption of about 25 mA.

Furthermore, if, in addition to the previously described autonomy-enhancing strategies, it is considered that dynamic monitoring does not need to be strictly continuous, but can instead be performed intermittently, for example, by acquiring data for 10 min every hour, which is sufficient for modal tracking problems, then the achievable autonomy can be significantly increased. Taking this scenario as an example and considering that the accelerometers modules installed on the deck consume approximately 10 µAh in sleep mode, while those on the stay cables consume about 50 µAh in the same circumstances, the previously reported autonomy values could be increased by a factor of approximately six. This would correspond to an autonomy of about 1.5 years for the deck sensors and 6.8 months for the stay cable sensors.

### 5.2. Development of Edge Computing Tools

In the case of dynamic measurements, the previously reported autonomy levels can only be achieved if the transmitted data are condensed into a reduced set of parameters, which requires the development and implementation of Edge Computing tools. In general, all sensors compute a set of parameters at the end of each 10-min acquisition period, such as battery charge and the maximum values of the measured variables in each direction, which typically do not represent significant computational challenges. However, the determination of the RMS value can be more demanding, as it involves squaring the signal values, which may lead to overflow errors in the presence of high-amplitude vibration peaks, particularly given the limited computational capacity of the microcontroller. This issue becomes even more critical when calculating higher-order parameters, such as the VDV (Vibration Dose Value). To address these challenges, the adopted strategy consists of performing the calculations incrementally, processing the data in smaller ranges and applying appropriate scaling before exponentiation. This approach is described in detail in reference [23].

Another task that must be performed locally at the sensor level is spectral analysis. In this work, an algorithm was developed to estimate the natural frequency of the stay cable without the need to transmit the complete time signal to the receiver. The proposed methodology, described in Section 6.1.2, involves the computation of the average spectrum of the signal, which is a particularly demanding task for local execution. To address this challenge, a dedicated subroutine was developed to compute the signal spectrum and store intermediate results in the microcontroller’s memory, enabling the extraction of the average spectrum at the end of the process [1].

## 6. Estimating Stay Cable Dynamics and Axial Forces

The implementation of the monitoring system was motivated by previous work conducted by the research group on the bridge’s stay cables, within the scope of a service aimed at characterizing their dynamic behavior and estimating the axial forces prior to the rehabilitation works [24]. In this regard, the present section focuses on performing this identification by leveraging experimental data collected over a one-month period, using the developed edge computing tools to automatically estimate axial forces directly at the sensor level. It is worth noting that this analysis focuses exclusively on the instrumented long cable No. 6, as described in Section 3.1.

### 6.1. Identification of the Natural Frequencies

#### 6.1.1. First Identification Based on APSD Maps

**Figure 18:**
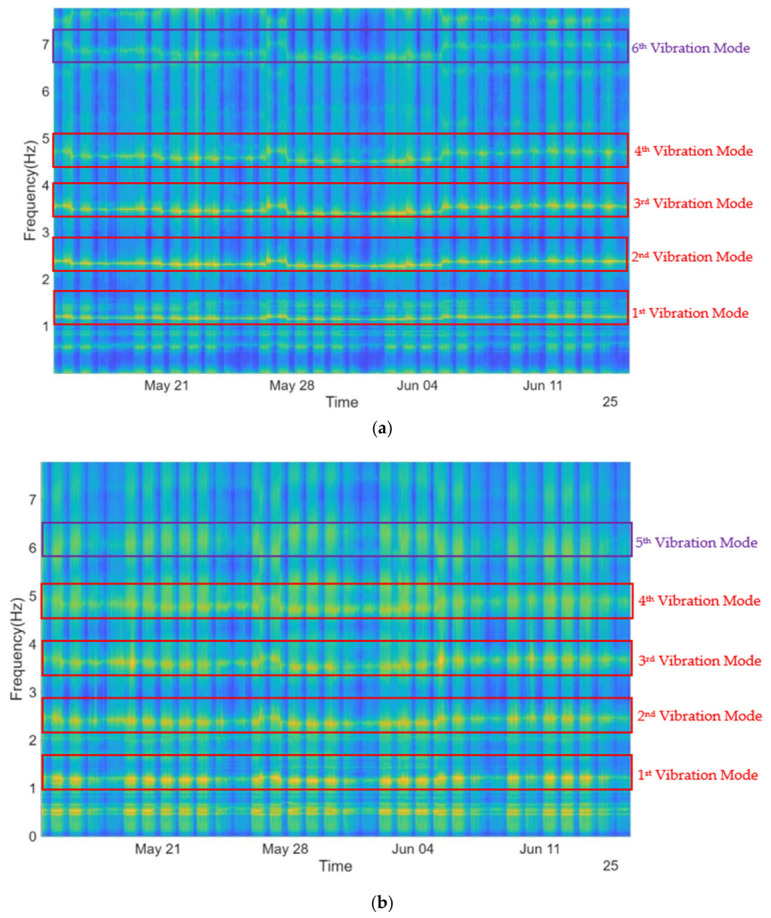
Spectral maps of the accelerometer ACC1: (**a**) Lateral direction; (**b**) transversal/vertical.

Figure 18 presents the maps of the Average Power Spectral Density (APSD) computed by the sensor and transmitted to the receiver at 10-min intervals. These maps enable the identification of the natural frequencies of the stay cable, which appear as horizontal bands of higher intensity. In both measurement directions (lateral and vertical/transversal), several natural frequencies can be identified, with the fundamental frequency located at approximately 1.2 Hz and higher-order modes appearing around 2.4 Hz, 3.6 Hz, 4.8 Hz, and 7 Hz. It can be observed that the natural frequencies exhibit variability over time, which can be immediately attributed to construction-phase effects, particularly those associated with the movement of the construction platform on the deck.

#### 6.1.2. Automatic Identification Based on Edge Computing

**Figure 19:**
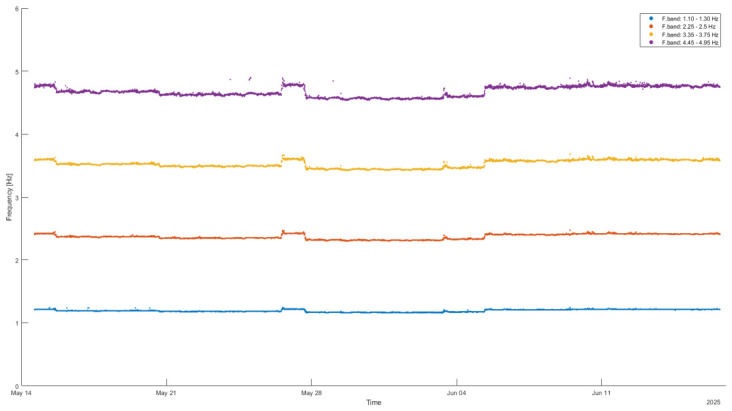
Identification of the first four natural vibration frequencies of the cable.

**Figure 20:**
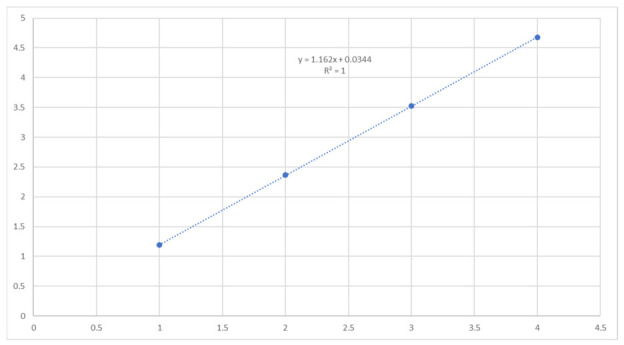
Progression of the average values of the identified frequencies.

The Edge Computing tools described in Section 5.2 can be used to estimate the natural frequencies of the cable, offering the advantages previously highlighted. The methodology adopted in this case study is based on the following points:

(a) For stay cables, it is well known that the natural frequencies are approximately integer multiples of the fundamental frequency, which facilitates the identification of frequency bands where these modes are expected to occur.
(b) Based on previous work [24], a set of frequency bands was defined.
(c) Since the sensor is installed on the flexible cable, the dynamic response of this element dominates over that of the overall structure, meaning that the spectral peaks within these bands are primarily associated with the cable dynamics rather than the global structural response, as can be seen in Figure 19 and Figure 20.
(d) By identifying the peak locations within the defined frequency bands, the natural frequencies of the cables can be determined.

**Figure 21:**
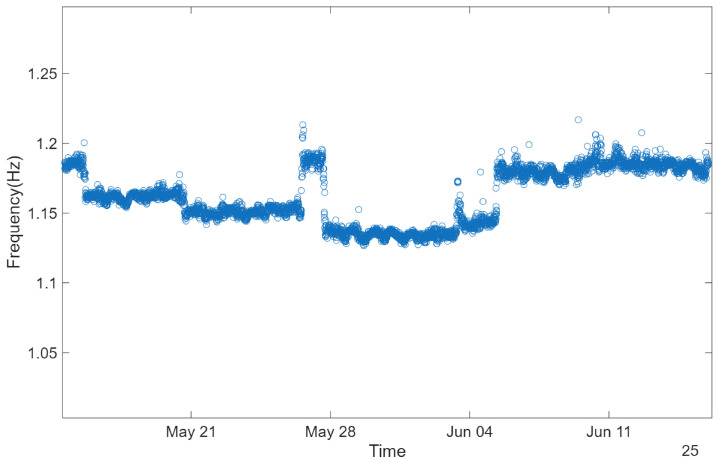
Fundamental frequency of the stay cable over time.

Figure 19 presents the results of this procedure, clearly showing the identification of the first four natural frequencies of the cable. Furthermore, assuming a linear progression of the natural frequencies, as illustrated in Figure 20 where the average values of the identified frequencies are plotted, the estimation of the fundamental frequency can be further refined by dividing a higher-order frequency by its corresponding mode number. This approach is illustrated in Figure 21, where the third natural frequency is used for this purpose.

The resulting graph shows sudden variations in the natural frequency of the cable, which are clearly associated with the movement of the construction sliding platform on the deck, as well as smaller daily fluctuations related to diurnal temperature variations.

### 6.2. Estimation of Axial Forces

The determination of axial forces in cables is critical for the analysis and design of cable-supported structures. Direct measurement of these forces is often challenging and costly. Alternatively, the dynamic properties of cables, particularly their vibration characteristics, can be used to indirectly estimate axial forces. In this context, factors such as bending stiffness, sag effects, and cable–deck interaction should be considered. It is well established that the influence of bending stiffness decreases for long cables, as is the case here [25]. However, sag effects and cable–deck interaction may become significant and can influence the accuracy of the estimated cable tension. These effects can be accounted for using advanced techniques, including approaches based on wave propagation and nonlinear behavior [26].

However, in the present study, an approximate estimation of the axial force is considered sufficient for comparison purposes, as this variable is directly provided by the construction company through direct measurements. To this end, the taut string theory offers a simplified framework that can be adopted. According to this approach, the cable is idealized as a taut string, neglecting the aforementioned effects. The natural frequencies of the cable, obtained from ambient vibration measurements, are then related to the axial force through the expression given in Equation (1), where *T* is the cable tension, $f_{n}$ is the *n*-th natural frequency of the cable, *m* is the cable mass per unit length, *l* is the cable length, and *n* is the mode number.

(1) $$T=4ml^{2}{\left(\frac{f_{n}}{n}\right)}^{2}$$

**Figure 22:**
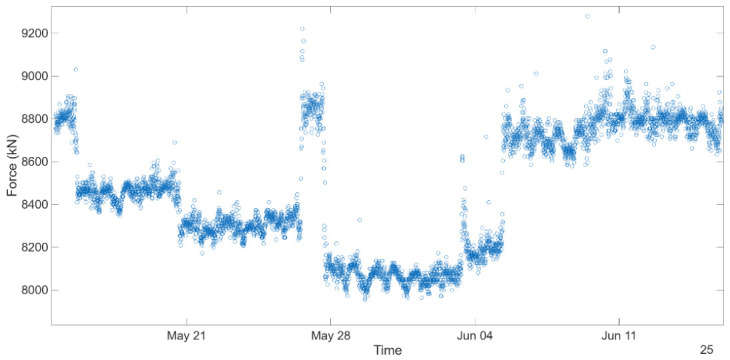
Scatter plot of cable force over time.

Accordingly, utilizing the data obtained from the description of the cables and their characteristics, summarized in Table 2, together with the frequency estimated according to Section 6.1.2, it was possible to estimate the axial forces by applying this method, which is depicted in Figure 22.

For comparison purposes, considering an average natural frequency close to 1.16 Hz, the tensile force was estimated at 8433 kN and tensile stress of about 515.8 MPa, which is in line with the estimates reported in previous studies [20,24]. In addition, a direct measurement of the cable force was performed after the stay-cable system substitution by Dywidag, which yielded a value of 8.575 kN, further corroborating the experimental estimations. This consistency reinforces the validity of the adopted methodology and provides confidence in the reliability of the experimental data as well as in the simplified assumptions inherent to the taut string model.

## 7. Identification of the Deck Dynamics

The acceleration signals recorded on the bridge structure by accelerometers ACC1 and ACC2 were used to estimate the structural dynamic behavior during the monitoring period, particularly regarding the evolution of the natural frequencies. The identification of vibration mode shapes was not performed, as it would require additional measurement points and is therefore beyond the scope of this study. Unlike what was done for the stay cable and given that the accelerometers were accessible at any time from the deck, data was collected locally on the SD cards and post-processed using Matlab v.R2025b subroutines.

**Figure 23:**
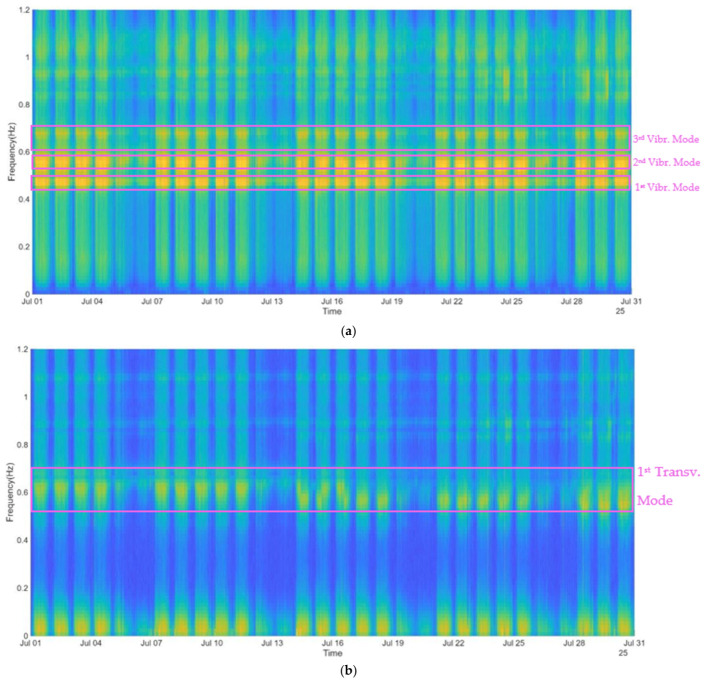
Time–frequency representation (ANPSD) of the bridge deck in two directions: (**a**) vertical direction and (**b**) lateral direction.

As a result, the spectral maps indicated in Figure 23, corresponding to both vertical and lateral directions measured by accelerometer ACC2, allowing us to identify several stable frequency components clearly associated with the natural frequencies of the structure. The first 3 frequencies are 0.45 Hz, 0.55 Hz and 0.7 Hz related to vertical vibrations modes, and the transverse frequency, more difficult to identify, can be estimated at 0.65 Hz approximately.

According to previous studies performed in 2020 (before the ongoing rehabilitation) those frequencies were identified at 0.50 Hz, 0.57 Hz and 0.725 Hz for vertical modes and 0.854 Hz as first transverse vibration mode [24]. At an even earlier date, in 1997, these frequencies were identified at 0.51 Hz, 0.60 Hz and 0.73 Hz, respectively, corresponding to the first 3 vertical vibration modes and 0.87 Hz to the first transverse direction mode [20]. However, taking into account that the actual measured frequencies were evaluated during the rehabilitation works where additional masses are present on the bridge (such as the sliding platform and scaffolding structures) it is expected that natural frequencies are lower in this stage compared to previous tests. For clarity, Table 3 summarizes the first natural frequencies evaluated over time.

## 8. Characterization of Temperature and Displacements Profiles

This section aims to characterize variables with lower dynamic variability, namely temperature and displacements. While temperature was measured continuously throughout the monitoring period, displacement measurements were only available for a limited time interval, as the sensors were installed in areas subject to frequent maintenance interventions, which compromised the reliability and continuity of the data. In the case of the displacement sensor installed at the expansion joint, data was collected for only one week. For the sensor located at the hinge, reliable data could not be obtained, reason why these measurements were not considered in the present analysis.

### 8.1. Temperature Measurements

**Figure 24:**
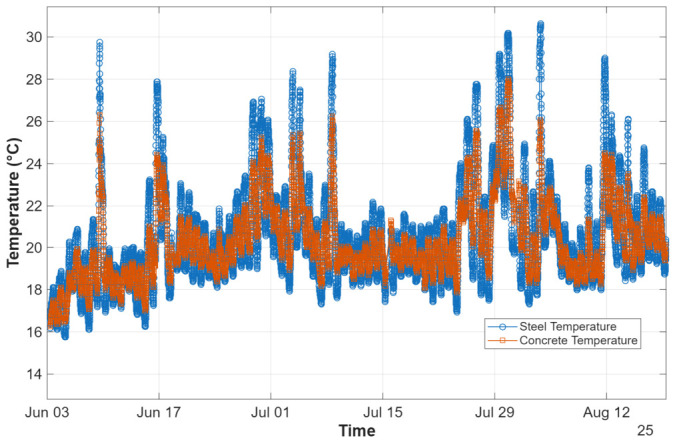
Temperature measurements registered during the monitoring program.

The temperatures were recorded over time for two different materials: steel and concrete, by using respectively T1 and T2. As shown in Figure 24, both sensors follow a similar pattern, reflecting the influence of environmental conditions in both materials. The sensor in the steel generally shows slightly higher temperature fluctuations compared to the sensor in the concrete, which is consistent with the higher thermal conductivity and lower thermal inertia of steel. Additionally, this sensor is installed on the steel beam that supports the deck, which is directly exposed to solar radiation, causing the expected temperature range to be greater. On the other hand, the temperature sensor installed in the concrete tends to exhibit a more inertial response of lower amplitude, as the material stores and releases heat more gradually. The peaks observed on certain days are likely related to periods of increased solar exposure, while the overall gradual increase in reference temperatures from May to August reflects the expected seasonal warming. Monitoring these temperature variations is essential to understand their potential impact on structural behavior, as temperature changes can influence material properties, boundary conditions and, consequently, the static and dynamic response of the bridge.

### 8.2. Displacements at the Expansion Joint

**Figure 25:**
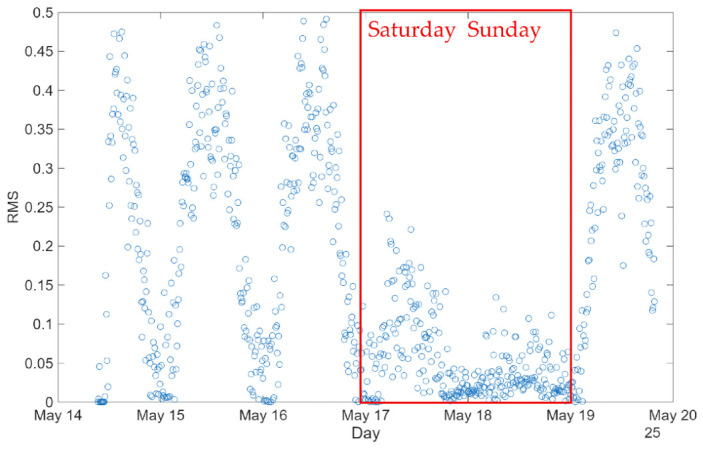
Weekly measurements: differences between weekdays and weekends.

Similarly to what was observed in the acceleration signals shown in Figure 25, where higher spectral energy is concentrated during daytime periods on working days, the RMS values of the displacements at the expansion joints exhibit the same trend. This increase in dynamic response can be clearly attributed to traffic loads, which induce higher vibration levels in the deck, as well as small pseudo-dynamic displacements at the expansion joints.

Figure 25 represents the RMS values of the displacements over a period of one week. It is worth mentioning that the RMS values refer to the dynamic component of the signal after detrending. In fact, during weekdays, the RMS response often reaches values approaching 0.45 mm. The higher the number of vehicles, the greater the displacements of the expansion joint caused by increased traffic loads.

In contrast, weekends (highlighted in red) show significantly lower RMS levels, mainly below 0.1 mm. This reduction reflects the considerable decrease in traffic demand during these periods, when both light and heavy vehicle traffic are substantially reduced.

### 8.3. Correlation Between Temperature and Expansion Joint Displacement

The displacement observed in the expansion joint of the bridge is directly influenced by temperature variations in the structure. Both steel and concrete structural elements undergo thermal expansion and contraction when subjected to temperature changes. Although their coefficients of thermal expansion are similar (in the order of 10^−5^/°C), even small variations in temperature can lead to significant cumulative displacements over the total length of the bridge.

**Figure 26:**
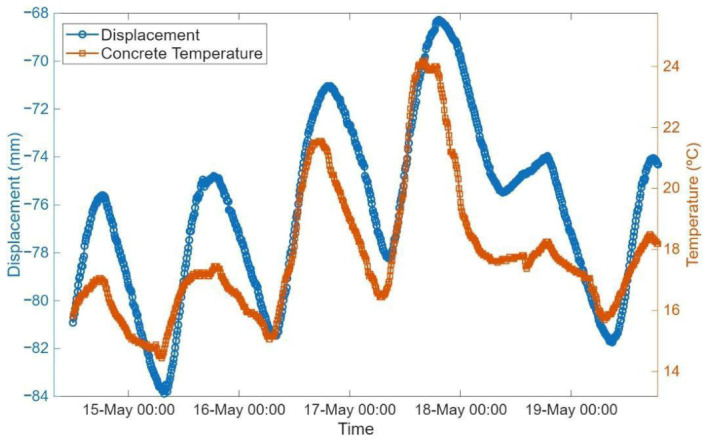
Displacement and concrete temperature over time.

Figure 26 illustrates the cyclical behavior of temperature and the corresponding joint movement. Progressive contraction is observed during the night, while expansion occurs during the day. Moreover, periods of thermal stability correspond to relatively steady displacement values, while rapid temperature changes are directly reflected in abrupt displacement shifts.

Overall, the data underscores the critical role of temperature-induced movements in joint behavior. The combination of displacement and temperature measurements provides clear evidence of the joint’s expansion and contraction cycles, demonstrating how thermal dynamics govern structural responses.

## 9. Conclusions

This paper describes the short-term (one-month) structural monitoring of a cable-stayed bridge undergoing rehabilitation works, using customized sensors, namely accelerometers, temperature sensors and displacement sensors and one reception station.

These sensors were strategically positioned on critical points of the bridge to capture its static and dynamic responses. The system was designed with a strong emphasis on autonomy, low energy consumption, and wireless communication, enabling remote data transmission to a reception station located off-site. In parallel, a digital platform with a database and graphical interface, which ensured the organization, processing and real-time visualization of the information collected was developed, which proved to be fundamental in increasing data accessibility and supporting the future integration of large-scale monitoring systems. The overall evaluation of the system performance is very positive, despite some sensor failures resulting from their installation in locations subject to frequent maintenance interventions, which compromised the reliability and continuity of the measurements.

Although such systems do not constitute a new insight into current advances in monitoring technologies, this paper provided important contributions by emphasizing strategies that enable customized systems to achieve a high degree of autonomy. In particular, it was shown that the selection of the microcontroller integrated into the sensor is a key factor in optimizing power consumption, being the consumption levels quantified for two different platforms. Furthermore, it was demonstrated that reducing data transmission and GPS operating time to short intervals has a limited impact on overall energy consumption. In this context, the sensor autonomy was quantified for the accelerometers used in both the stay cable and the deck. It was also highlighted that, in dynamic monitoring applications, if the system does not require strictly continuous operation but can instead operate intermittently, the achievable autonomy can be significantly increased by taking advantage of sleep modes.

Additionally, a methodology was proposed for the automatic and real-time estimation of axial forces in cables. This approach relies on the use of innovative edge computing tools, combined with the taut string theory as a simplified modelling framework. Through this method, abrupt variations in the cable’s natural frequency associated with the movement of the construction platform were identified, as well as daily fluctuations related to temperature effects on the cable. The estimated axial forces were compared with direct measurements provided by Dywidag, showing very good agreement and thereby validating the proposed methodology.

Finally, preliminary results from the processing of accelerometer signals on the deck, as well as temperature and displacement measurements at the expansion joint, were presented. It was concluded that traffic loads significantly influence both the vibration levels of the bridge and the movement of the expansion joints. The effect of temperature on joint movement was also analyzed. However, due to the limited duration of the available data, no quantitative relationship could be established.

## Figures and Tables

**Table 1:** Nomenclature of devices.

Nomenclature	Device
ACC	Accelerometer
DSP	Displacement Sensor
T	Temperature Sensor
R	Reception Station

**Table 2:** Characteristics of the cables.

Parameters	Values
Area	163.5 cm^2^
*m*	149.37 kg/m
*l*	102.42 m

**Table 3:** Bridge deck natural frequencies identified over time.

	Vertical Vibration Modes			Transversal Vibration Mode
	1st Mode	2nd Mode	3rd Mode	1st Mode
2025	0.45 Hz	0.55 Hz	0.70 Hz	0.65 Hz
2020	0.50 Hz	0.57 Hz	0.725 Hz	0.854 Hz
1997	0.51 Hz	0.60 Hz	0.73 Hz	0.87 Hz

## Data Availability

The data presented in this study are available on request from the corresponding author due to (specify the reason for the restriction).

## Appendix A

**Table A1:** Receptor File Type description.

Device	Parameter	Time Delay [s]
DSP1	Battery voltage $\left[V\times 10^{2}\right]$	15
	Average Value $\left[mm\times 10^{2}\right]$	
	RMS of the measurements $\left[mm\times 10^{2}\right]$	
	Maximum Relative Value $\left[mm\times 10^{2}\right]$	
	Minimum Relative value $\left[mm\times 10^{2}\right]$	
DSP2	Battery voltage $\left[V\times 10^{2}\right]$	30
	Average Value $\left[mm\times 10^{2}\right]$	
	RMS of the measurements $\left[mm\times 10^{2}\right]$	
	Maximum Relative Value $\left[mm\times 10^{2}\right]$	
	Minimum Relative value $\left[mm\times 10^{2}\right]$	
T1 E T2	Battery voltage $\left[V\times 10^{2}\right]$	45
	Temperature 1 $\left[{}^{\circ}C\times 10^{2}\right]$	
	Temperature 2 $\left[{}^{\circ}C\times 10^{2}\right]$	
ACC2	Battery voltage $\left[V\times 10^{2}\right]$	60
	Box Temperature $\left[{}^{\circ}C\times 10^{2}\right]$	
	Apx $\left[{m/s}^{2}\times 1000\right]$	
	Apy $\left[{m/s}^{2}\times 1000\right]$	
	Apz $\left[{m/s}^{2}\times 1000\right]$	
	Armsx $\left[{m/s}^{2}\right]$	
	Armsy $\left[{m/s}^{2}\right]$	
	Armsz $\left[{m/s}^{2}\right]$	
ACC3	Battery voltage $\left[V\times 10^{2}\right]$	75
	Box Temperature $\left[{}^{\circ}C\times 10^{2}\right]$	
	Apx $\left[{m/s}^{2}\times 1000\right]$	
	Apy $\left[{m/s}^{2}\times 1000\right]$	
	Apz $\left[{m/s}^{2}\times 1000\right]$	
	Armsx $\left[{m/s}^{2}\right]$	
	Armsy $\left[{m/s}^{2}\right]$	
	Armsz $\left[{m/s}^{2}\right]$	
ACC1	Battery voltage $\left[V\times 10^{2}\right]$	90
	Box Temperature $\left[{}^{\circ}C\times 10^{2}\right]$	
	Apx $\left[{m/s}^{2}\times 1000\right]$	
	Apy $\left[{m/s}^{2}\times 1000\right]$	
	Apz $\left[{m/s}^{2}\times 1000\right]$	
	Armsx $\left[{m/s}^{2}\right]$	
	Armsy $\left[{m/s}^{2}\right]$	
	Armsz $\left[{m/s}^{2}\right]$	
	Frequency band 1 $\left(0.6–1.8\;\mathrm{Hz})\;[Hz\times 10^{3}\right]$	
	Frequency band 2 $\left(1.8–3.0\;\mathrm{Hz})\;[Hz\times 10^{3}\right]$	
	Frequency band 3 $\left(3.0–4.2\;\mathrm{Hz})\;[Hz\times 10^{3}\right]$	
	Frequency band 4 $\left(4.2–5.4\;\mathrm{Hz})\;[Hz\times 10^{3}\right]$	
	Frequency band 5 $\left(5.4–6.6\;\mathrm{Hz})\;[Hz\times 10^{3}\right]$	
	Frequency band 6 $\left(6.6–7.8\;\mathrm{Hz})\;[Hz\times 10^{3}\right]$	
	Frequency band 7 $\left(7.8–9.0\;\mathrm{Hz})\;[Hz\times 10^{3}\right]$	
	Frequency band 8 $\left(9.0–10.2\;\mathrm{Hz})\;[Hz\times 10^{3}\right]$	
	Frequency band 9 $\left(10.2–11.4\;\mathrm{Hz})\;[Hz\times 10^{3}\right]$	
